# Supplementary material for: Reconciling Mining with the Conservation of Cave Biodiversity: A Quantitative Baseline to Help Establish Conservation Priorities
Source: PLoS One. 2016 Dec 20;11(12):e0168348. doi: 10.1371/journal.pone.0168348 (PMC5173368; doi:10.1371/journal.pone.0168348)
Supplement: S1 Dataset — (ZIP) [file pone.0168348.s002.zip › Taxa/Serra Norte/SN_2013/N5S/Lista N5S-37.pdf]

CAVIDADE N5S-0037

Classe	Ordem	Fam/Outros	Gên/Outros	Espécie	S-U	U-S	Zon.
Annelida	Oligochaeta			jovem	7	8	P/A
Arachnida	Acari	(Parasitengonina)		sp.2		X	P
Arachnida	Acari	(Parasitengonina)		sp.9		X	P
Arachnida	Acari	Cunaxidae		sp.1	X		P
Arachnida	Acari	Eupodidae		sp.1	X		P
Arachnida	Acari	Laelapidae		sp.72	X		P
Arachnida	Acari	Laelapidae		sp.73	X		P
Arachnida	Acari	Rhagidiidae		sp.4		X	P
Arachnida	Acari	Sphaerolichidae		sp.1		X	P
Arachnida	Acari	Trigynaspida		sp.1		X	P
Arachnida	Acari	Trombiculidae		sp.39		X	P
Arachnida	Acari	Trombidiformes		sp.1		X	P
Arachnida	Acari	Trombidiformes		sp.49	X		P/A
Arachnida	Acari	Trombidiidae		sp.15	X		P/A
Arachnida	Amblypygi	Charinidae	*Charinus*	sp.1	1		E
Arachnida	Amblypygi	Charinidae	*Charinus*	sp.1		2	P
Arachnida	Amblypygi	Phrynidae	*Heterophrynus*	*longicornis*	3		A
Arachnida	Araneae	Caponiidae		sp.1	X	X	E/P/A
Arachnida	Araneae	Corinnidae	*Creugas*	sp.1		2	P
Arachnida	Araneae	Drymusidae	*Drymusa*	*spelunca*	X	X	P
Arachnida	Araneae	Ochyroceratidae	*Ochyrocera*	sp.1	X	X	E/P
Arachnida	Araneae	Ochyroceratidae	*Ochyrocera*	sp.3		X	P
Arachnida	Araneae	Ochyroceratidae	*Speocera*	sp.1	X		P
Arachnida	Araneae	Pholcidae	*Mesabolivar*	sp.1	X		E
Arachnida	Araneae	Theraphosidae		jovem	1		P
Arachnida	Araneae	Theridiidae	*Theridion*	sp.1	X		P
Arachnida	Araneae	Theridiosomatidae	*Plato*	sp.1	X	X	E/P
Arachnida	Opiliones	Cosmetidae	*Roquettea*	*singularis*	1		P
Arachnida	Opiliones	Escadabiidae		sp.1	X	X	E/P
Arachnida	Opiliones	Escadabiidae		sp.2	X	X	P
Arachnida	Opiliones	Stygnidae	*Protimesius*	*gracilis*	2		P
Arachnida	Palpigradi	Eukoeneniidae	*Allokoenenia*	sp.1	X	X	P
Arachnida	Palpigradi	Eukoeneniidae	*Eukoenenia*	sp.1	X		P
Arachnida	Pseudoscorpiones	Bochicidae		sp.1		X	P
Arachnida	Pseudoscorpiones	Chernetidae	*Spelaeochernes*	sp.1	X		P
Arachnida	Pseudoscorpiones	Chthoniidae	*Pseudochthonius*	sp.2		X	P
Chilopoda	Geophilomorpha	Geophilidae		sp.3		2	P
Chilopoda	Scolopendromorpha	Cryptopidae	*Cryptops*	jovem		1	P
Chilopoda	Scolopendromorpha	Scolopocryptopidae	*Newportia*	sp.1	1		E
Chilopoda	Scutigeromorpha	Pselliodidae	*Sphendononema*	jovem		1	P
Diplopoda	Polydesmida	Chelodesmidae		sp.3		7	P
Diplopoda	Polydesmida	Fuhrmannodesmidae		jovem	X		A
Diplopoda	Polydesmida	Pyrgodesmidae		sp.1	X	X	P/A
Entognatha	Collembola	Cyphoderidae		sp.1		X	P
Entognatha	Collembola	Isotomidae		sp.1	X	X	P/A
Entognatha	Collembola	Paronellidae		sp.4	X	X	P/A
Entognatha	Collembola	Sminthuroidea		sp.1	X	X	P
Entognatha	Collembola	Sminthuroidea		sp.2	X	X	P
Entognatha	Diplura	Campodeidae		sp.1	X		E/P

Insecta	Blattodea	Blattellidae		sp.1	3	2	P/A
Insecta	Blattodea	Blattellidae		sp.5		1	P
Insecta	Coleoptera	Carabidae	*Coarazuphium*	sp.n.2	X		
Insecta	Coleoptera	Chrysomelidae		sp.4	X		E/P
Insecta	Coleoptera	Dytiscidae		sp.11C	X	X	P/A
Insecta	Coleoptera	Dytiscidae		sp.12A	X		A
Insecta	Coleoptera	Staphylinidae		sp.3		X	P
Insecta	Diptera	Chaoboridae		sp.	X		P
Insecta	Diptera	Culicidae	*Culex*	sp.	X	X	P
Insecta	Diptera	Psychodidae	Phlebotominae	sp.	X	X	E/P/A
Insecta	Heteroptera	Dipsocoroidea		sp.	X	X	P
Insecta	Heteroptera	Reduviidae	Emesinae	sp.		1	P
Insecta	Heteroptera	Reduviidae	*Panstrongylus*	jovem	1		P
Insecta	Heteroptera	Reduviidae	Reduviinae	sp.	4	3	P/A
Insecta	Homoptera	Cixiidae		jovem	X	X	E/P
Insecta	Hymenoptera	Diapriidae		sp.2		X	P
Insecta	Hymenoptera	Diapriidae		sp.4		X	P
Insecta	Hymenoptera	Formicidae	*Camponotus*	sp.2		X	P
Insecta	Hymenoptera	Formicidae	*Hypoponera*	sp.1		X	P
Insecta	Hymenoptera	Formicidae	*Hypoponera*	sp.2	X		E
Insecta	Hymenoptera	Formicidae	*Pachycondyla*	sp.1		X	P
Insecta	Hymenoptera	Formicidae	*Pachycondyla*	sp.2	X	X	P/A
Insecta	Isoptera	Rhinotermitidae	*Heterotermes*	sp.	X		P
Insecta	Isoptera	Termitidae	*Nasutitermes*	sp.		X	P
Insecta	Lepidoptera			jovem	X	X	P
Insecta	Orthoptera	Phalangopsidae	*Paraclodes*	sp.	1		P
Insecta	Orthoptera	Phalangopsidae	*Phalangopsis*	sp.	64	240	P/A
Insecta	Psocoptera	Epipsocidae	*Epipsocus*	sp.2	X		E/P
Insecta	Thysanura	Nicoletiidae		sp.1	X		E
Gastropoda	Pulmonata	Subulinidae		sp.2		X	P
Gastropoda	Pulmonata	Systrophiidae	*Happia*	sp.		X	P
Malacostraca	Isopoda	Dubioniscidae		sp.1	X		P
Malacostraca	Isopoda	Scleropactidae	*Circoniscus*	*buckupi*	X	X	P
Pauropoda	Tetramerocerata			sp.	X		P
Symphyla		Scutigerellidae		sp.	X	X	P
Amphibia	Anura	Strabomantidae	*Pristimantis*	cf. *fenestratus*	10	1	P
Mammalia	Chiroptera	Emballonuridae	*Peropteryx*	*kappleri*		5	E/P
Mammalia	Chiroptera	Furipteridae	*Furipterus*	*horrens*	5	10	E/P/A
Mammalia	Chiroptera	Phyllostomidae	*Trachops*	*cirrhosus*	4		P/A
